# Supplementary material for: Gal-2 Increases H3K4me3 and H3K9ac in Trophoblasts and Preeclampsia
Source: Biomolecules. 2022 May 15;12(5):707. doi: 10.3390/biom12050707 (PMC9139023; doi:10.3390/biom12050707)
Supplement: Supplementary file 1 [file biomolecules-12-00707-s001.zip › biomolecules-1689025-supplementary.pdf]

biomolecules

MDPI

Supplementary Table S1. Gestational age and sex of the babies of healthy and PE affected pregnancies.

Controls			PE						
Week of Gestation	Sex of the Baby	Maternal Age	Week of Gestation	Sex of the Baby	Weight of the Baby (g)	APGAR	Maternal Age	Proteinuria (mg/dL)	Blood Pressure (mmHg)
40 + 3	male	17	39 + 0	male	3260	10/10/10	-	300	170/100
40 + 3	male	25	36 + 4	male	3250	8/10/10	31	100	-
40 + 2	female	39	35 + 0	female	1555	8/10/10	-	-	180/100
40 + 2	female	31	34 + 6	-	-	-	-	-	-
40 + 1	female	33	34 + 3	male	2100	9/10/10	38	51,4	176/100
39 + 6	male	30	34 + 3	male	1980	10/10/10	38	51,4	176/100
39 + 6	female	23	34 + 1	male	1704	9/10/10	-	-	-
39 + 1	male	35	34 +1	male	2000	7/9/10	-	300	190/100
39 + 1	male	32	32 + 5	-	-	-	29	-	-
39 + 0	male	32	31 + 1	Female	1370	7/9/9	40	156	195/95
38 + 3	female	35	29 + 5	-	-	-	-	-	-
38 + 0	male	22	-	-	-	-	-	-	-
35 + 4	male	34	-	-	-	-	-	-	-
